# Natural Language Processing and Graph Theory: Making Sense of Imaging Records in a Novel Representation Frame

**DOI:** 10.2196/40534

**Published:** 2022-12-21

**Authors:** Laurent Binsfeld Gonçalves, Ivan Nesic, Marko Obradovic, Bram Stieltjes, Thomas Weikert, Jens Bremerich

**Affiliations:** 1 Clinic of Radiology & Nuclear Medicine University Hospital Basel University of Basel Basel Switzerland

**Keywords:** radiology, deep learning, NLP, radiology reports, imaging record, temporal referrals, date extraction, graph theory, health care information system, resource planning.

## Abstract

**Background:**

A concise visualization framework of related reports would increase readability and improve patient management. To this end, temporal referrals to prior comparative exams are an essential connection to previous exams in written reports. Due to unstructured narrative texts' variable structure and content, their extraction is hampered by poor computer readability. Natural language processing (NLP) permits the extraction of structured information from unstructured texts automatically and can serve as an essential input for such a novel visualization framework.

**Objective:**

This study proposes and evaluates an NLP-based algorithm capable of extracting the temporal referrals in written radiology reports, applies it to all the radiology reports generated for 10 years, introduces a graphical representation of imaging reports, and investigates its benefits for clinical and research purposes.

**Methods:**

In this single-center, university hospital, retrospective study, we developed a convolutional neural network capable of extracting the date of referrals from imaging reports. The model's performance was assessed by calculating precision, recall, and F1-score using an independent test set of 149 reports. Next, the algorithm was applied to our department's radiology reports generated from 2011 to 2021. Finally, the reports and their metadata were represented in a modulable graph.

**Results:**

For extracting the date of referrals, the named-entity recognition (NER) model had a high precision of 0.93, a recall of 0.95, and an F1-score of 0.94. A total of 1,684,635 reports were included in the analysis. Temporal reference was mentioned in 53.3% (656,852/1,684,635), explicitly stated as not available in 21.0% (258,386/1,684,635), and omitted in 25.7% (317,059/1,684,635) of the reports. Imaging records can be visualized in a directed and modulable graph, in which the referring links represent the connecting arrows.

**Conclusions:**

Automatically extracting the date of referrals from unstructured radiology reports using deep learning NLP algorithms is feasible. Graphs refined the selection of distinct pathology pathways, facilitated the revelation of missing comparisons, and enabled the query of specific referring exam sequences. Further work is needed to evaluate its benefits in clinics, research, and resource planning.

## Introduction

Radiology departments generate tremendous amounts of reports every day. Narrative radiology reports are the primary communication medium between radiologists and referring physicians, thus playing a central role in patient care and containing a large variety of health care information [1,2]. From 1996 to 2010, image study volume for computed tomography (CT) and magnetic resonance imaging (MRI) increased by 280% to 380% [3]. Radiology embraced digital workflows and electronic information transfer to referring colleagues early on, which virtually eradicated analog data in this field [4]. This early commitment provides enormous quantities of digitalized reporting data containing interpretative image descriptions. However, the extraction of this information is hampered because unstructured reports are poorly computer-readable [5]. Semantic reports contain valuable information at a granular level (eg, multiple temporal referrals) that can be evoked for the overall report or specific findings in multiple document locations. This multilocular information cannot easily be determined on a whole document level [6].

Natural language processing (NLP) is one solution to the problem of extracting specific information from the plethora of free-text radiology reports. NLP is defined as the analysis of linguistic data, most commonly in the form of textual data, using computational methods [7-13]. NLP has evolved from rule-based to machine learning algorithms [14-20], deep learning being a subset of the latter that applies multilayer neural networks [21,22]. Its capability to automatically extract structured information has been described in many medical research settings [23-29]. Especially in radiology, there are numerous instances where it has demonstrated excellent text mining performances, including the detection of incidental findings and recommendations [30-32], actionable findings [33], specific findings [34-41], quality assessment of reports [42,43], and the generation of curated data sets [44-49]. 

The quantitative accumulation of radiology reports per patient over the years has led to a highly interconnected network of exams. Modern picture archiving and communication systems (PACS) represent the different exams as a list sorted by their acquisition date. Most systems can highlight the previous exams of roughly the same region in the study description to the user. This type of comparative visualization does not consider multiregional studies or often-encountered findings at the margins of the acquired field of view. It does not foreground the dates to which the radiologist compared his findings in the report. This last part especially is a significant shortcoming for clinicians reviewing patient history. They have to read every report carefully to see to which point in time the radiologist compared tumor progress, for example, or if the images from an external institute were available to the radiologist at the exact time when reading the follow-up exam.

One crucial connection in this context is dated referrals to prior exams. The good practice guidelines for radiological reporting from the European Society of Radiology [50] and the 2020 revised American College of Radiology practice parameter for communication of diagnostic imaging findings emphasize the need for comparison with previous investigations, including the date of previous reports and mentioning the absence of previous imaging. By using comparison studies, radiologists make more observations, gain confidence in their interpretation, and provide more diagnoses [51-55]. One study found that the diagnostic accuracy, sensitivity, and specificity in mammography increased as the false-positive rate decreased [56]. Various recent studies relied on NLP techniques to extract the temporality of measurements in imaging reports (ie, attributing an observation on the current or prior exam) [39-62]. However, to the best of our knowledge, no methods that extract every referring date from semantic radiological texts have been researched. Moreover, no studies in the literature have focused on the overall temporal indexing of the report assessed, in most instances, by the radiologist at the beginning of the report.

One solution to displaying connections between a multitude of different reports is graph representation. Graph theory defines graphs as a set of properties stored in nodes connected by edges, which represent a relationship between the connected nodes [63,64]. A review paper from 2020 found that graphs, as defined by graph theory, are hardly used to represent patient data in a clinical context; in the literature review, only 11 papers matched the description [65]. 

This study aimed to develop a novel and concise visualization framework of related reports.

To this end, we applied a self-designed NLP algorithm capable of extracting the referencing dates from unstructured radiology reports on all the reports generated for 10 years at a university hospital. This information was an essential input for a relational graph in which the nodes represent the radiology reports with their associated metadata and the dated referrals are their connecting edges. Finally, we investigated the potential benefits of such a graph representation and storage for clinical and research purposes.

## Methods

### Ethics Approval

Institutional review board approval and the requirement for informed consent were waived (institutional review board: Ethikkommission Nordwest- und Zentralschweiz) since no patient identifiers were used. Collected data consisted of plain text from radiology reports and randomized metadata, neither of which could be tracked back to radiologists, individual patients, nor referring clinicians.

### Data Set Acquisition and Description

We extracted all radiology reports from January 2011 to December 2021 as well as a selection of their associated Digital Imaging and Communications in Medicine (DICOM) metadata (ie, randomized patient ID, modality type, body region, study date) from the hospital database. All reports were written in German and derived from all the imaging modalities (ie, ultrasound, radiography, mammography, x-ray angiography, CT, MRI, nuclear medicine exams, and positron emission tomography [PET]-CT). The reports were a mix of unstructured free-text reports and standardized templates, either containing subheadings for distinct organs with prewritten normal findings (eg, CT chest-abdomen) or checklists for standardized reporting features (eg, Liver Imaging Reporting and Data System for liver MRI). The broad structure of the reports was usually divided into 5 sections: medical history, medical question, examination protocol, radiological finding, and impression. 

Every radiology exam had a predefined body region and modality type in its DICOM metadata. There were 14 body regions and 9 modalities (see Multimedia Appendix 1).

### Construction of a Temporal Reference Extraction Algorithm 

#### Data Selection for Training

We randomly selected 5187 reports from the previously extracted radiology reports.

#### Data Annotation

An internally developed data annotation tool, “xtag,” was used. A second-year medical resident (LBG) manually labeled 5187 reports with 5 classes indicating the temporal reference (Table 1). The annotation classes “date,” “today,” “yesterday,” and “no previous” were applied on the text sequence level (ie, annotating sequences of numbers or words). The annotation class “missing” was applied at the document level and was exclusive, meaning that no other annotation could be applied. On the other hand, “date,” “today,” “yesterday,” and “no previous” could be applied multiple times per report. To assess the necessity of a second reading, a fifth-year medical resident in radiology (TW) annotated 100 randomly selected reports. This process yielded 100% agreement among readers. Considering the simplicity of the task and based on this result, we refrained from a second reading of the whole data set.

**Table 1:** Annotated classes and their defined meaning.

Class	Meaning
Date	Precise numerical date referring to a comparative exam; any numerical or partially numerical format was accepted.
Today	Non-numerical temporal reference to a comparative study done on the same day as the actual report (ie, any literal expression meaning today)
Yesterday	Non-numerical temporal reference to a comparative study done on the day before the actual report (ie, any literal expression meaning yesterday)
No previous	Explicit statement that no comparable previous exams are available
Missing	No mention of a comparative study

#### Data Format

The training pipelines required the annotations to conform to the IOB2 format [66,67]. The predictions were also produced in the same format (further technical information can be found in Multimedia Appendix 2 [5,68-72]).

#### Algorithm Training and Testing

We excluded 2392 reports from the annotated data set, as they did not contain temporal links. We split the data into a training/validation data set of 2646 reports (94.6%) and an independent test data set of 149 reports (5.4%). We estimated that 5% for an independent and second test data set is a valid representation, as we verified the algorithm's robustness using the 5-fold cross-validation [73]. We also considered the low output variability of the problem to be solved. We used the Spacy sentencizer to text into sentences before training. We then used the ktrain library to produce a bidirectional long short-term memory (LSTM) [74] model starting with pretrained fastText word embeddings [75] (for details, see Multimedia Appendix 2). We applied various rule-based date extraction algorithms on the predicted date sequences to extract as many dates as possible. The non-numerical classes today and yesterday were converted into a numerical format using the date of the referring report as a reference. The dates missing the year specification were assigned the same year as the referencing report. The prediction was ignored if the day or month was missing. A grid search algorithm tested different combinations of learning rates and batch sizes to find the near optimal parameters for our training algorithm. A 5-fold cross-validation [76] of the training data set with 20% of the reports as validation in each round was performed to evaluate the model's performance on large independent data sets. The data set split into folds was done at the report level. The model was tested on the independent test data set in a final evaluation step**.** The following performance evaluation metrics were used to assess the trained model's quality: precision, recall, and *F*_1_-score [77].

#### Extraction of the Referenced Modality and Body Region 

The referenced modality was extracted using a simple rule-based approach. After extracting the temporal references from the report, the algorithm searched for a mention of the modality in the sentence with the date reference. The previous report's body part was derived from its metadata and was assumed to be the same as the referencing report's body region. 

#### Graduation of the Predicted Link's Confidence

We graduated the prediction's confidence as follows: (1) date, modality, and body part; (2) date and modality; (3) date and body part; (4) date. This confidence graduation was established as a link property, in which 1 was the most confident and 4 was the least confident. The link was discarded if it was impossible to generate it based on these 4 principles. This approach permitted narrowing and increasing the accuracy of the referenced reports if more than one exam was acquired on the referenced date. 

### Algorithm Application and Data Extraction on the Complete Data Set

The preparatory steps for extraction of the temporal information by the trained model were the same as for the training part. The result of the model's application to all the reports from 2011 to 2021 was a per-token table with labels for each token in the IOB2 format. Predictions that did not comply with the IOB2 format were removed. 

### Populating the Graph Database

The graph database system used was Neo4j (Version 4.4.). All the reports and a selection of their associated metadata from 2011 to 2021 were imported via the py2neo library. The metadata consisted of the exam's acquisition date, name, modality, and body region, as well as its randomized patient ID. The reports and their metadata were assigned to the vertices, and unidirectional edges from referencing reports to referenced reports were created. We assigned 3 properties to the edges: The first consisted of the inferred class called “reference class,” the second showed the extracted string, and the third displayed the prediction's confidence.

### Interactive Exploration of the Graph 

The assessment of the potential benefits of patient data visualization in a graph was explored interactively. The aim was to offer, at one glance, a well-ordered overview of the patient's imaging history with the related reports; enable comparison to previous exams; and represent the desired pathology pathway in a concise way (eg, oncological or postoperative follow-up imaging). In addition, it reveals to the clinician and the radiologist at what point in time the radiologist made his or her comparison. The user should be able to restrict his or her search to individually adaptable filters in the report's metadata (eg, body region, modality type, report date, or keywords in the reports' text). Another important feature would be to provide precisely filtered examinations in a concise order, in which every exam has its precisely defined position in a sequence. A final goal was to assess missed comparisons to previous exams, which was hoped to be achieved visually by spotting the missing link in the graph and by self-designable search algorithms.

## Results

### Data Set 

**Figure 1:**
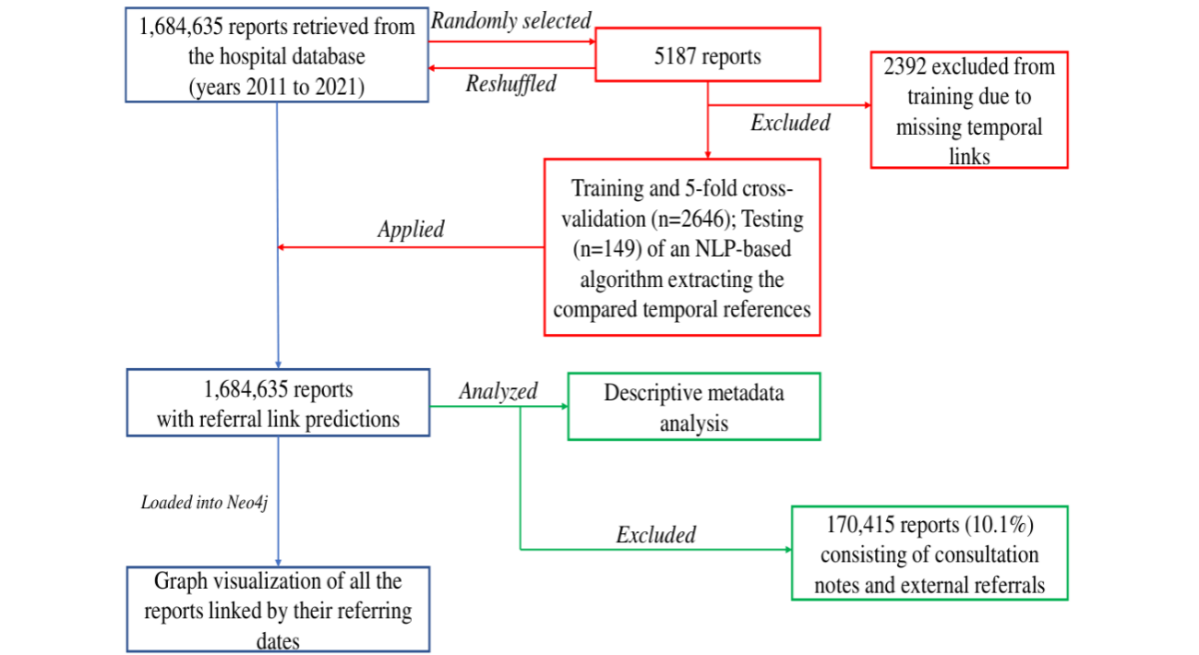
Study flowchart of 1,684,635 patient reports retrieved from the hospital database (2011 to 2021). NLP: natural language processing.

In total, 1,684,635 reports from 264,655 distinct patients were extracted. We excluded 170,415 (10.1%) reports from the metadata analysis because they consisted of consultation notes and external referrals (detailed count in Multimedia Appendix 3).  Figure 1 shows the detailed methodical flowchart.

### Annotation Distribution 

A total of 7860 annotations were applied to 5187 reports from 2011 to 2019. Class distribution of the training data set was as follows: 44% date reference, 27% no previous comparative exam, 23% missing temporal link and 6% referral class “today.” We removed the semantic referencing class “yesterday” from our data set as there were not enough training samples (34/5187, 0.7%). 

### Temporal Information Extraction Algorithm

#### Hyperparameter Optimization 

The algorithm's output yielded an optimal learning rate of 1e-2 and a batch size of 1024. The random state was fixed for reproducibility. The maximal number of training epochs was limited to a never reached limit of 30. 

#### Training and Testing

The stagnation in the validation performance of 3 epochs was targeted for the early stopping. During training, the model is stored after each epoch. After the completion of the training process, the best-performing epoch weights were used for the final model. The same procedure was used for all the steps in which training was involved. After 5-fold cross-validation (results in Multimedia Appendix 4)**,** the algorithm's performance was tested on the previously unused test data set (Table 2).

**Table 2:** Test results on 149 previously unused reports.

Variable	Precision (95% CI)	Recall (95% CI)	*F*_1_-score (95% CI)
Date	0.93 (0.89-0.93)	0.9 (0.86-0.93)	0.93 (0.91-0.94)
No previous	0.94 (0.95-0.97)	0.98 (0.96-0.98)	0.96 (0.93-0.98)
Today	0.76 (0.73-0.88)	0.85 (0.79-0.90)	0.83 (0.79-0.93)
Micro average	0.93 (0.91-0.94)	0.92 (0.90-0.95)	0.94 (0.89-0.93)
Macro average	0.86 (0.84-0.95)	0.91 (0.87-0.95)	0.91 (0.80-0.94)
Weighted average	0.93 (0.91-0.94)	0.93 (0.90-0.94)	0.94 (0.91-0.95)

### Temporal Referencing Analysis 

**Figure 2:**
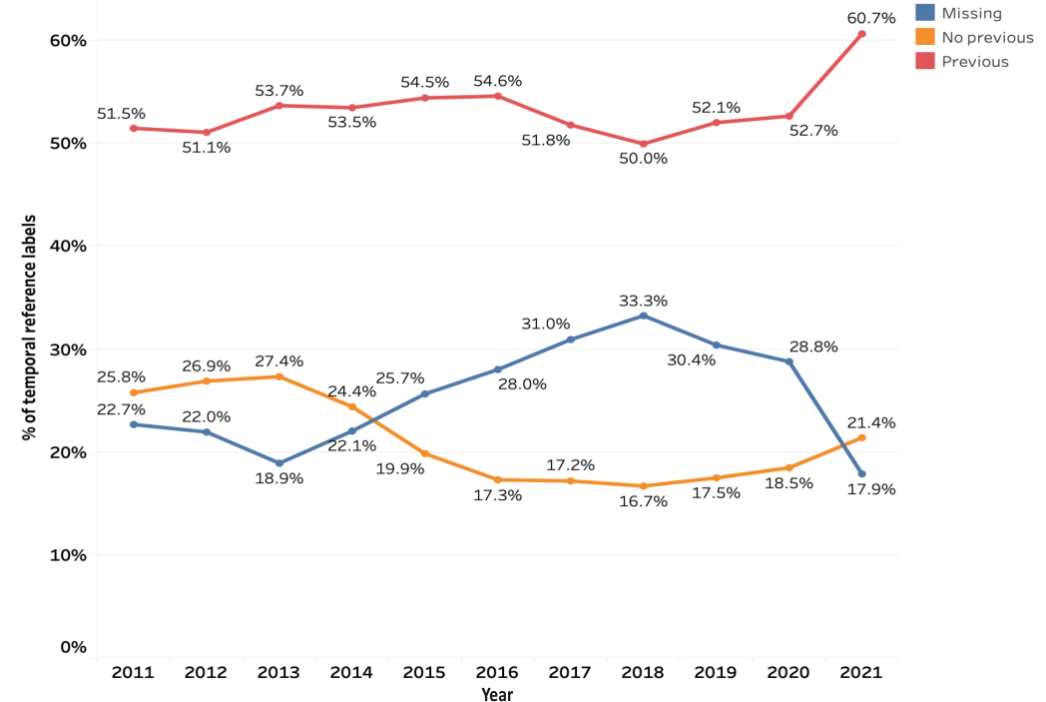
Temporal reference of the reports (n=1,514,220) over the years.

A temporal reference to comparable exams was mentioned in 53.3% (656,852/1,232,297), explicitly stated as not available in 21.0% (258,386/1,232,297), and omitted in 25.7% (317,059/1,232,297) of the reports. Variability over the years was asserted (Figure 2). The modalities with the least amount of missing references were mammography (41,197/545,636, 7.6%), PET/CT (1850/18,500, 10.3%), and CT (278,286/2,399,017, 11.6%). On the other hand, angiography (33,924/40,872, 83.2%) and ultrasound (94,080/254,270, 37.2%) had the most missing references (Table S4 in Multimedia Appendix 5). The body regions with the lowest amount of missing references were trunk (3072/39,639, 7.8%), breast (5727/70,617, 8.1%), and thorax (25,646/276,060, 9.3%). On the other hand, the heart (19,030/26,090, 72.9%) and neck (14,716/23,230, 63.4%) regions had the most missing links (Table S5 in Multimedia Appendix 5)**.** Modalities primarily referred to the same modality except for angiography referring to plain radiographs in 39.8% (1790/4503), PET/CT referring to MRI in 45.1% (456/1013), and nuclear medicine exams referring to CT in 33.9% (3500/10294; Table S6 in Multimedia Appendix 5). Every body region predominantly referred to the same body region. The most extreme example was “breast,” which was referenced in 99.0% (59,619/60,221) of the cases by the other breast studies.

### Analysis of the Median Time Period of the Referencing Reports

The median period between referencing reports from 2011 to 2021 was determined in days, per modality (Table 3) and body region (Table 4). The most extended periods were found in mammography (372 days) and the corresponding body region breast (370 days). The shortest periods were observed in plain radiograph reports (19 days) and the thorax region (10 days). 

**Table 3:** Median time period between referencing reports (n=757,249) per modality.

Modality	Time period (days), median (Q1-Q3)	IQR	*P* value
Computed radiography	19 (2-118)	116	<.001
X-ray angiography	35 (7-137)	130	.048
Computed tomography (CT)	42 (3-231)	228	<.001
Magnetic resonance	65 (3-344)	341	.002
Nuclear medicine	114 (8-440)	432	.06
PET^a^/CT	129.5 (30-366)	336	<.001
Ultrasound	344 (24-386)	362	.002
Mammography	372 (352-722)	368	.03

^a^PET: positron emission tomography.

**Table 4:** Median time period between referencing reports (n=757,249) per body region.

Body region	Time period (days), median (Q1-Q3)	IQR	*P* value
Thorax	10 (2-156)	154	.01
Upper extremity	11 (1-48)	47	.01
Abdomen	35 (4-237)	233	.006
Spine	35 (3-207)	204	<.001
Pelvis	39 (3-146)	143	.001
Lower extremity	42 (6-136)	130	.01
Head	65 (2-364)	362	<.001
Trunk	89 (34-196)	162	.03
Heart	125 (8-378)	370	=.40
Whole body	128 (8-427.3)	419.3	<.001
Neck	182 (29-395)	366	.045
Breast	370 (348-550)	202	.009

### Exploration of Imaging Records in a Graph

#### General Overview 

**Figure 3:**
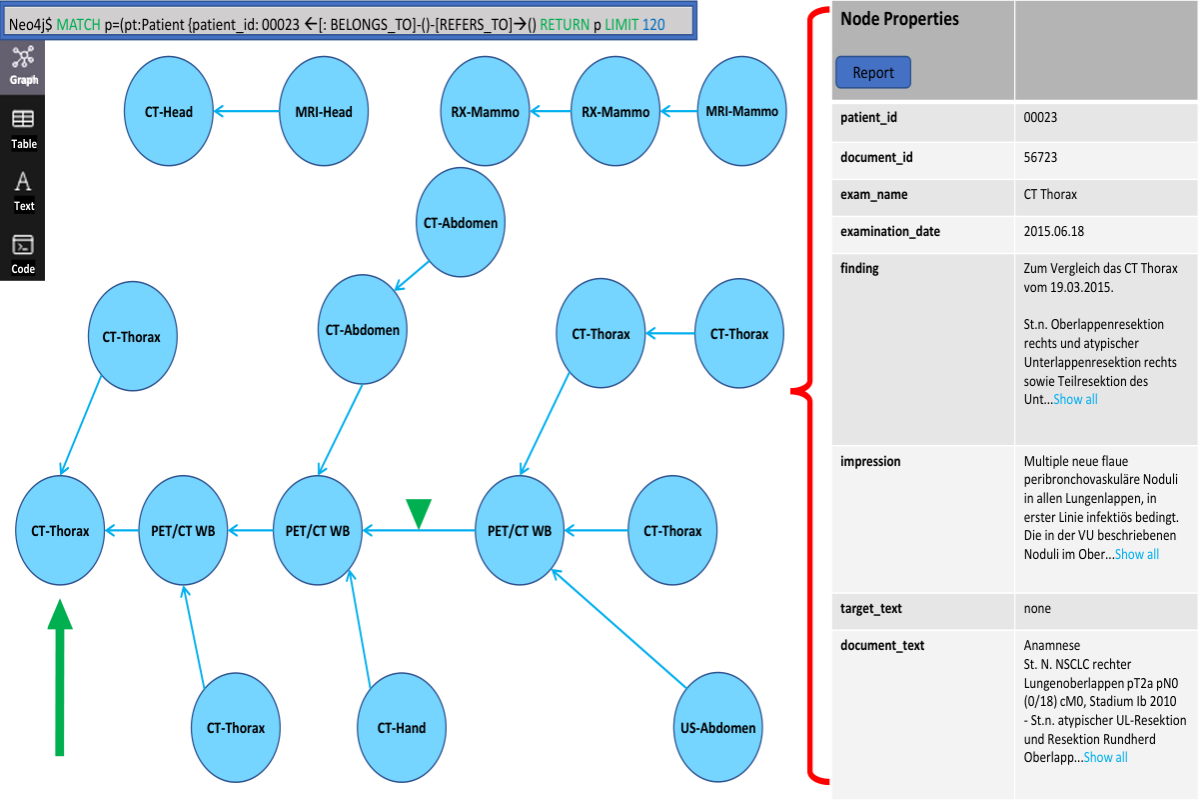
Single patient case example (lung cancer with cancer-related studies) including the full user interface in which the blue box in the top left is the query interface; the blue nodes contain the examination name, represent all the imaging studies stored in the picture archiving and communication system (PACS), and are ordered from oldest on the left to newest on the right; the connecting blue arrows represent their referral links; and a node’s metadata (examination name, acquisition date, text of the selected and referenced reports, and finding and impression sections) appears on the right side when the node is clicked on. CT: computed tomography; MRI: magnetic resonance imaging; PET: positron emission tomography; RX: x-ray; US: ultrasonography; WB: whole body.

All the imaging reports and metadata from 2011 to 2021 were successfully loaded into a directed graph. The blue nodes represented the different patient reports labeled with their examination name (eg, CT-chest or MRI-head), and the connecting links were their automatically extracted referral dates. The interface was individually adaptable (eg, the user could freely position the nodes as desired, and the colors of the individual components and displayed metadata were customizable). The total number of distinct patient reports could be selected at the beginning of any query. This view permitted a rapid visual assessment of the earliest comparison exam (Figure 3).

#### Multiparametric Filtered Representations 

**Figure 4:**
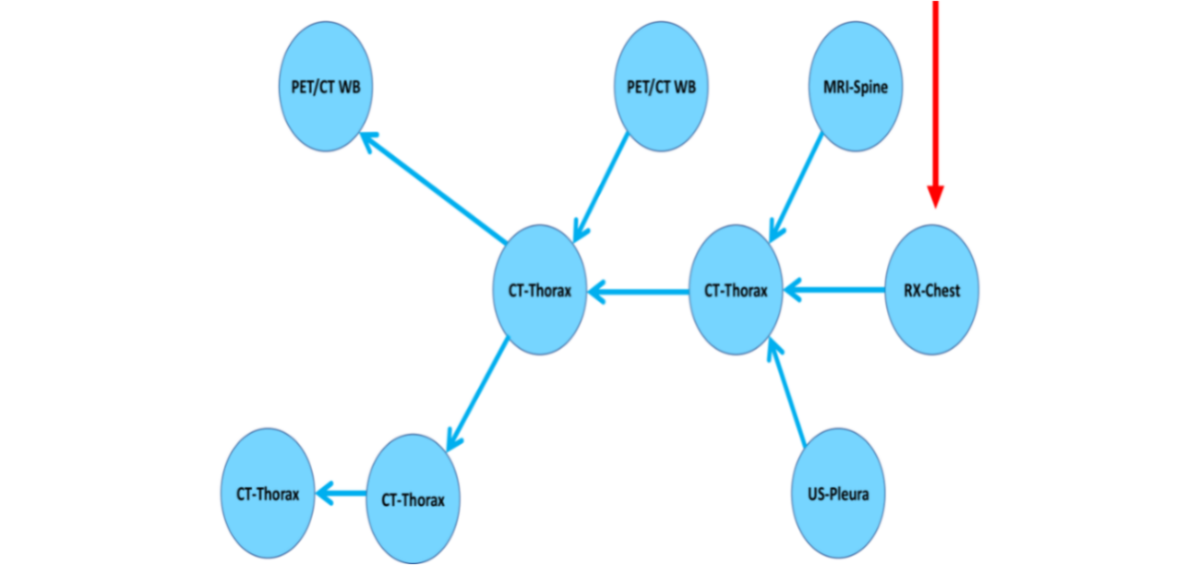
Filtered representation of related studies as a partial screenshot within the user interface, in which all the related prior exams appear at one glance by clicking on the last node referring to a lung cancer (red arrow). Although the user selected the most recent study, clicking on every other node in this network would have resulted in the same view. CT: computed tomography; MRI: magnetic resonance imaging; PET: positron emission tomography; US: ultrasonography; WB: whole body.

Narrowing the reports down to the most relevant and thus facilitating visualization are of utmost relevance with the high number of exams per patient. By clicking on the node of interest, the user could opt to display solely the linked reports (visualized in Figure 4). Another possible method of restricting the view and looking for specific findings was a search filter related to the associated metadata and specific words in the report's text. One possible concept would be to look for specific exams with no previous reference and a defined pathologic condition as a keyword in the report's text, which would speed up the selection of the first exam associated with this condition.

#### Specific Exam Sequence Selection

**Figure 5:**
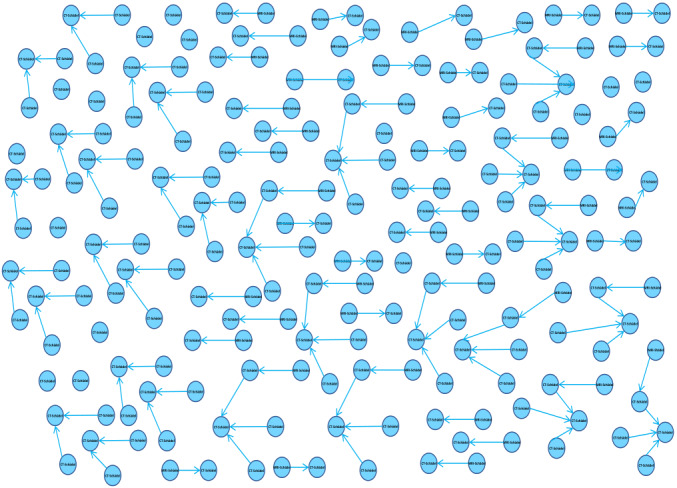
Specific exam sequence selection as a partial screenshot within the user interface, in which we used the query field to randomly select 300 reports (blue nodes) of head computed tomography (CT) referenced by a head magnetic resonance image (MRI) that was acquired no longer than 3 days later and contained the keyword “infarct” in the impression field.

**Figure 6:**
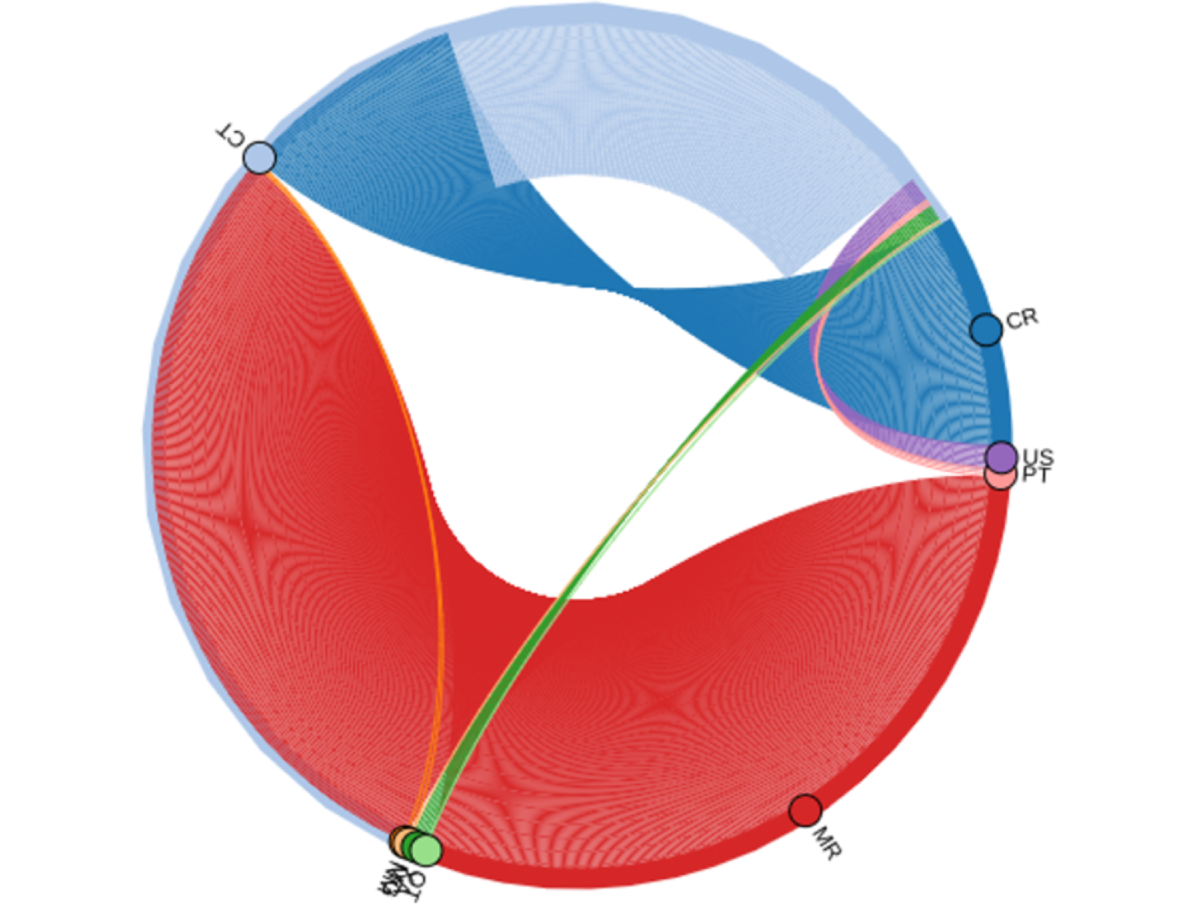
Chord diagram representing the connections between modalities in the head region referencing a head computed tomography (CT) image (light blue rim) during the 7 days after its acquisition. The size of the arc is proportional to the number of referenced reports. Most referring reports are head magnetic resonance images (MRIs), followed by other head CT images. CR: computed radiography; MR: magnetic resonance; NM: nuclear medicine; OT: other; PT: positron emission tomography; US: ultrasound.

Selecting highly customizable sequences of referring exams with specific metadata attributes (eg, chest x-ray followed by chest CT) was possible. This can be refined, for example, with a period restriction or restricted time interval between the related exams (Figures 5 and 6).

#### Visual and Filter-Aided Detection of Missing Comparative Connections

**Figure 7:**
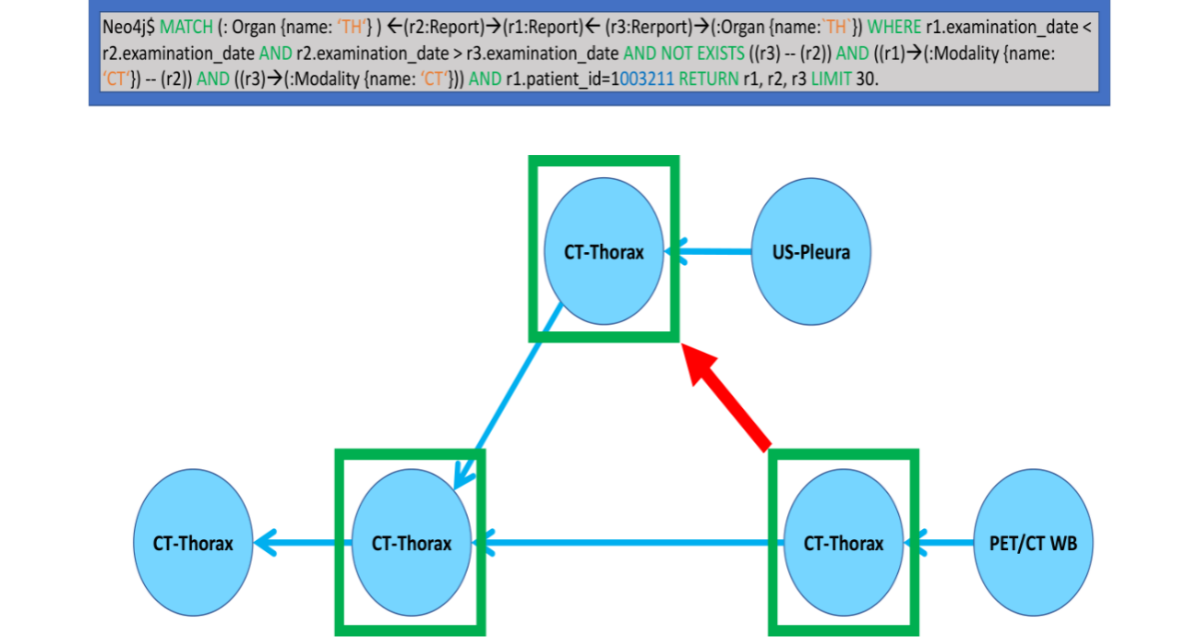
Single patient case example illustrating a missing temporal reference (red arrow) between subsequent reports (blue nodes, ordered by the earliest acquisition on the left to the latest on the right) of computed tomography (CT) studies of the thorax (green boxes). It is easy to detect a suspected missed link between the earlier CT-Thorax report at the top was not referenced by the later CT-Thorax report at the bottom right as well as via the search queries exposing temporal inconsistencies in the referrals (blue box at the top). PET: positron emission tomography; US: ultrasonography; WB: whole body.

Selective queries with sequential filters and graph visualization permitted a rapid assessment of situations in which referral links were missing (Figure 7). This feature was helpful when preceding comparative exams had been overlooked due to the poor list-like appearance of exam history in PACS or radiology information systems as well as when previous external images were imported into the PACS after the acquisition and reading of the following exam.

## Discussion

### Principal Findings

As shown in this paper, representing imaging records in a directed graph is feasible. Connecting them via their referring dates improved visualization of related imaging pathways and detected missed exam comparisons. We also showed that automated extraction of referring dates from written radiology reports using a deep learning–based NLP algorithm, the groundwork needed to create the representation, is feasible and achievable with high significance (*F*_1_-score of 0.94).

Considering the extraction of concepts of temporality using NLP, our method can be compared with a publication from 2019 by Bozkurt et al [60]. Their main focus was extracting measurements and their core descriptors, among other things, their temporal context, for which they used rule-based NLP with predefined regular expressions. They solely focused on 2 temporal aspects (ie, current or prior), and their pipeline had a high *F*_1_-score of 0.85. Our approach uses a date-extracting LSTM. It focuses on all the referring dates in a written report, including the ones without a precise measurement, for example, the lesions that cannot be measured due to an amorphous configuration or the overall comparison date of the report. In addition, our algorithm has the crucial and unique advantage of detecting the explicit absence and missingness of a comparative exam from written text. Furthermore, we extracted every date of comparison from the report, thus permitting a comminuted and precise linkage for constructing a general graph.

Our approach, however, has the main disadvantage of not attributing the comparison date to specific findings or measurements, which will slow down the focused review of specific entities in complex patient histories. Another disadvantage of our more granular extraction method is the high complexity of the task, which consecutively increases its dependency on correctly spelled referencing dates. Following this logic, omitted or wrongly chosen dates would have a greater impact on the integrity of the machine learning model and the graph in addition to the effect of varying writing habits or report templates between different institutions or radiologists. Although the reporting guidelines favor precisely dated comparisons, the radiologist does not always explicitly write the exact date of the compared finding in the text. As this omission mostly happens in comparison to the most recent report, which would be mentioned at the beginning of the report as the last referenced report, our method covers the majority of these cases. These aspects may render the overall applicability of our model more complex and susceptible to smaller errors than the temporality extraction algorithms developed so far.

In 2006, Lakhani et al [78] explored, in their large-scale database analysis of 1.8 million reports, how often radiologists compared with prior studies using a SQL approach. They found that 42.5% of reports completely omitted any reference to previous studies, 38.7% mentioned a comparison, and no relevant comparison was explicitly pointed out in only 18.8%. Although not entirely comparable, as they focused on a purely semantic approach of referring information extraction, it provides a good approximation, because if the reports contained phrases hinting toward a comparison, the date of the compared exam was most probably mentioned. In our study, reports referenced the date of a comparable exam (53%), explicitly stated that there was no previous exam (21%) more often, and were less prone to miss the referring link (26%). The best year for indicating temporal references was 2021, with only 17.9% of reports missing a reference, down from 30.4% in 2019 and 28.8% in 2020. This tendency toward more temporal referrals could result from the increased emphasis on comparison exam consultation and report structuring in current reporting guidelines and digitalization, with many previous studies easily accessible. However, these percentages of prior exam consultation based on the written references in radiology reports are most probably underestimates. Haygood et al [79] concluded in their study from 2018 that the assumption that an older radiologic image or medical document was not consulted during radiologic interpretation merely because it is not cited in the report was not valid. This causes medicolegal issues for the reader. Radiologists were found negligent by juries for failure to compare a new chest radiograph with all previous chest radiographs [80]. Without written proof, this gets more difficult to defend. Another relevant aspect is the different extraction approaches. Our sentence-based named-entity recognition system analyzed data on a granular level, thus not missing single dates meant for comparison in parenthesis or other dates without a clear semantic indication of referral like the SQL approaches required. 

Studies analyzing errors in the radiology reporting process emphasized the importance of comparing findings [52-56]. The good practice guidelines from the European Society of Radiology [50] and the 2020 revised American College of Radiology practice parameter for communication of diagnostic imaging findings support this affirmation. Kim and Mansfield [55] found that 5% of all errors in radiology resulted from failure to consult prior radiographic studies that could have led to the correct diagnosis. However, a critical review of the previous radiologists' findings or impressions should prevail when comparing previous exams. One must be careful not to follow an incorrect path; this error, called “satisfaction of report,” accounted for 6% of all errors reported in radiology in the study by Kim and Mansfield [55]. The widespread availability of previous exams in modern PACS renders an excuse for failing to compare findings with prior exams obsolete. The automatic selection of comparative exams offered by modern PACS is inherently biased because it primarily considers the locoregional aspect, thus losing focus on multiregionality. For example, a CT of the cervical spine or shoulder may be overlooked as a potential comparison source when evaluating apical lung masses, or abdominal radiographs when interpreting hips. The same logic applies to clinicians or radiologists reviewing the imaging history of a given finding, especially in oncology, which has many multiregional studies and findings.

These complex considerations call for a well-arranged and organized visualization system. Poor usability and hampered visualization of patient data reduce the motivation of thoroughly reviewing them, which remains a challenge in health care and is associated with increased error rates due to missing pertinent details, user fatigue, and frustration [78,79]. A study from 2022 analyzing the impact of intensive care unit clinical information systems showed that poor interface design and visual representations are major sources of dissatisfaction among users [80]. Our explorations indicate that grouping related exams together in a graph could help improve this fundamental and increasingly pressing user-friendliness issue. 

We hope that, by reinforcing the radiologist's organizing role and improving the case overview by replacing the list appearance of imaging history, he or she will tend to omit the referring links less often, thus minimizing comparison error. Another critical aid is the improved detection of omitted connections in situations in which, for example, previously acquired external scans were loaded into the PACS after reading the following exam. This would be of great value for the subsequent physicians reviewing the imaging history. Temporal referrals in a report prove to the reader that the radiologist has not forgotten to compare a specific finding. This is a valuable asset, considering that a finding's relevance is often determined by its temporal course. For example, lung nodules, brain atrophy changes, or vascular aneurysms showing no dynamic changes over a long period are less alarming, especially in infants and older adults, for whom noninvasive imaging follow-ups are favored over invasive medical investigations. Optimizing visualization with a graph representation could save time as well as decrease unnecessary exams and radiation exposure for patients.

In specialized medicine, clinicians are more focused on specific regions or findings. Manually filtering out the irrelevant exams adds work and a source of potential error (eg, an orthopedic surgeon is more inclined to investigate images implying the healing process of a fracture or a neurologist the exams related to cerebral or spinal findings). Our graph enables the user with an exam of interest to select all the related studies and to omit, if desired, all the unrelated reports, thus substantially and instantly reducing the number of studies to be reviewed.

Our system can assist quality control and review of guideline adherence by rapidly filtering out selectable sequences of exams (eg, CT performed after an x-ray) refined by the possibility of restricting the search for an interstudy period. This highly customizable review based on the reports' metadata could also help research projects. For example, when evaluating the features of a brain lesion over time, one could filter out all the reports in the database in which the finding is described in the report text; these reports will then be shown, if desired independently of the patient, with their respective related reports. This approach rapidly and intuitively speeds up an otherwise fastidious query, offering the researcher a follow-up and quick method for taking the measurement steps on the associated images. The quantitative and qualitative predictions as well as the period of the related following radiology exams could be of great value for clinical management purposes, permitting optimal prediction of the necessary human and material resources.

### Limitations

Our study has several limitations. The main limitation was that the analysis was based on a single tertiary care university hospital and depended strongly on our reporting customs. Second, reports were labeled by only 1 reader (a second-year resident). Given the low grade of complexity in labeling the referencing dates and the 100% agreement in a subset of 100 reports, we refrained from a second reading of the whole data set. The more challenging task of determining the comparative study was done during the reporting process by at least one board-certified radiologist. Third, there were insufficient samples to train the non-numerical referencing dates expressing “yesterday.” This should be addressed in future work. One solution could be to use active learning algorithms prioritizing the model's most uncertain predictions. Fourth, there was a lack of external validation. Also, to our knowledge, there is no comparable study in the literature. Nevertheless, the methodology should be reproducible in other radiology department setups to allow for future comparison. To this end, we have also made the codebase that allows for internal testing available (Multimedia Appendix 2). Fifth, the focus here was on the feasibility of an entire pipeline, including extraction and representation. Thus, we did not thoroughly evaluate its clinical usefulness but, instead, illustrated the potential usefulness in several use cases.

### Future Prospects

The high performance of our NLP-based model at processing immense amounts of free-text data underlines its potential for future research projects. The process of filtering out comparative studies could be accelerated substantially, which could greatly benefit the development of image detection–based and NLP-based algorithms. The concept of a related graph database could optimize the engineering and designing of other medical software tools in radiology by improving visualization and user-friendliness, accelerating data selection in research projects, and enhancing quality control and clinical review processes. An important amelioration could be the connection of the dates to the specific findings or measurements to which they are referring. Furthermore, it could enable resource planners to separately predict the necessary human and material resources. A significant asset of these databases is the easy-to-implement expansions (eg, integration of pathology reports or associated images). By giving users the possibility of correcting and adding links, it would be conceivable to create a continuously self-improving algorithm.

### Conclusion

We established a proof of concept of an NLP-based algorithm capable of accurately extracting the dates of referrals on a granular level from unstructured radiology reports. We successfully generated customizable graphs of referring radiology reports, in which multiple filters may freely be applied, providing a well-arranged visual overview. This type of visualization permitted new possibilities for querying specific exam sequences, facilitated the detection of missed comparisons by the radiologist, and offers health care professionals a wide range of review opportunities. The radiologist's awareness and motivation for the comparative aspect of his or her findings could be increased, and his or her worth for clinicians could be augmented by not solely providing information but also actively helping to organize it. Further work is needed to expand its features and evaluate its definite benefits in day-to-day clinical practice. 

## Appendix

Multimedia Appendix 1 Defining body regions and modality types.

Multimedia Appendix 2 Data format and machine learning.

Multimedia Appendix 3 Distinct count in the whole data set.

Multimedia Appendix 4 Five-fold cross-validation results.

Multimedia Appendix 5 Metadata analysis of the temporal references.

## Abbreviations

CT: computed tomography
DICOM: Digital Imaging and Communications in Medicine
LSTM: long short-term memory
MRI: magnetic resonance imaging
NLP: natural language processing
PACS: picture archiving and communication system
PET: positron emission tomography
