# Supplementary material for: Natural Language Processing and Graph Theory: Making Sense of Imaging Records in a Novel Representation Frame
Source: JMIR Med Inform. 2022 Dec 21;10(12):e40534. doi: 10.2196/40534 (PMC9813822; doi:10.2196/40534)
Supplement: Multimedia Appendix 1 [file medinform_v10i12e40534_app1.docx]

There were 14 body regions and 9 modalities (see Table S1). The modality “nuclear medicine” consisted of a mix of all the examinations performed in nuclear medicine (ie, scintigraphy, SPECT, and thyroid echography, except for PET/CT, which got a distinct category). The DICOM metadata designation of “other” in modality as well as “not applicable” and “not characterized” in body region were not considered in the descriptive metadata analysis because they consisted of patient consultation notes and external referrals (eg, echocardiographic reports done by cardiologists stored in a separate RIS).

**Table S1.** Body regions and modalities.

| Body regions | Modalities |
| --- | --- |
| Abdomen | X-ray |
| Pelvis | CT^a^ |
| Heart | MRI^b^ |
| Thorax | Mammography |
| Trunk | Nuclear medicine |
| Head | Other |
| Neck | PET^c^/CT |
| Upper extremity | Ultrasonography |
| Lower extremity | X-ray angiography |
| Spine |  |
| Mamma |  |
| Whole body |  |
| N/A^d^ |  |
| Not specified |  |

^a^CT: computed tomography.

^b^MRI: magnetic resonance imaging.

^c^PET: positron emission tomography.

^d^N/A: not applicable.
