# Supplementary material for: Natural Language Processing and Graph Theory: Making Sense of Imaging Records in a Novel Representation Frame
Source: JMIR Med Inform. 2022 Dec 21;10(12):e40534. doi: 10.2196/40534 (PMC9813822; doi:10.2196/40534)
Supplement: Multimedia Appendix 2 [file medinform_v10i12e40534_app2.docx]

## Data Format

The data was extracted in sequences and converted into formats that could be used with various pipelines, tools, or algorithm implementations. The training algorithm consumed data in the inside, outside, beginning 2 (IOB2) format, which is a slight modification of the chunking IOB format. Our self-made pipeline, which utilized pandas and Spacy libraries, was used to transform the character level annotation into IOB2.

## Machine Learning

Temporal information extraction fits to the subtask of information extraction called named entity recognition (NER) [5,76]. Sequence labeling is an algorithmic pattern recognition dealing with assigning categorical labels to text sequences. A common problem solved by sequence labeling is part-of-speech (POS) tagging [77] or grammatical tagging, which categorizes the sentence parts with similar grammatical properties (eg, nouns, verbs, and adjectives are just a few possibilities).

Bidirectional LSTM recurrent neural networks, with an optional conditional random field (CRF) [78] layer, successfully resolve scheme embeddings on word and character levels. CRFs consider the dependency between each state and all the input sequences, overcoming the label bias using a global normalizer.

With this in mind, we used the ktrain library [79] and trained a bidirectional LSTM model with pretrained fastText word embeddings for the German language. The fastText uses a continuous bag of words (CBOW) and skip-gram to train word representation models. We refrained from using a CRF due to incompatibility with the TensorFlow 2 library. The algorithms used in this study are openly available on GitHub: To access them, please visit [80].
