# Supplementary material for: Natural Language Processing and Graph Theory: Making Sense of Imaging Records in a Novel Representation Frame
Source: JMIR Med Inform. 2022 Dec 21;10(12):e40534. doi: 10.2196/40534 (PMC9813822; doi:10.2196/40534)
Supplement: Multimedia Appendix 3 [file medinform_v10i12e40534_app3.docx]

In total, 1,684,635 reports from 264,655 distinct patients were extracted. We excluded 170,415 (10.1%) reports from the metadata analysis because they consisted of consultation notes and external referrals. Table S2 illustrates the count of the different body regions and modality types.

**Table S2.** Descriptive metadata analysis data set consisting of 1,514,220 radiology department reports from 2011 to 2021.

| Body region and modality | | 2011 (n=110,624) | 2012 (n=113,869) | 2013 (n=117,528) | 2014 (n=126,439) | 2015 (n=127,098) | 2016 (n=134,369) | 2017 (n=155,478) | 2018 (n=157,945) | 2019 (n=159,637) | 2020 (n=148,053) | 2021 (n=163,180) |
| --- | --- | --- | --- | --- | --- | --- | --- | --- | --- | --- | --- | --- |
| **Abdomen (n=155,289)** | | | | | | | | | | | | |
|  | X-ray (n=8457) | 1771 | 1517 | 1281 | 862 | 659 | 507 | 396 | 476 | 335 | 344 | 309 |
|  | CT^a^ (n=39,077) | 2535 | 2770 | 3083 | 3270 | 3236 | 3622 | 3856 | 4039 | 4462 | 3900 | 4304 |
|  | MRI^b^ (n=12,278) | 513 | 574 | 670 | 871 | 967 | 1032 | 1245 | 1356 | 1608 | 1563 | 1879 |
|  | NM^c^ (n=2016) | 195 | 239 | 203 | 176 | 162 | 181 | 225 | 167 | 158 | 166 | 144 |
|  | PET^d^-CT (n=11) | 0 | 0 | 0 | 0 | 0 | 0 | 0 | 0 | 0 | 0 | 11 |
|  | US^e^ (n=71,510) | 3210 | 3884 | 3893 | 4845 | 6153 | 6797 | 7943 | 7597 | 8299 | 8273 | 10,616 |
|  | X-angio^f^ (n=21,940) | 1547 | 1501 | 1715 | 1967 | 1976 | 1956 | 2086 | 2085 | 2227 | 2373 | 2507 |
|  | Total | 9771 | 10,485 | 10,845 | 11,991 | 13,153 | 14,095 | 15,751 | 15,720 | 17,089 | 16,619 | 19,770 |
| **Breast (n=72,861)** | | | | | | | | | | | | |
|  | X-ray (n=7) | 7 | 0 | 0 | 0 | 0 | 0 | 0 | 0 | 0 | 0 | 0 |
|  | MRI (n=4740) | 244 | 253 | 326 | 427 | 439 | 481 | 497 | 453 | 472 | 539 | 609 |
|  | Mammography (n=42,668) | 3467 | 3666 | 3861 | 3783 | 3806 | 3955 | 3668 | 3897 | 3940 | 4192 | 4433 |
|  | NM (n=24) | 0 | 0 | 0 | 0 | 0 | 0 | 0 | 2 | 8 | 7 | 7 |
|  | US (n=25,420) | 1995 | 2149 | 2169 | 2074 | 2117 | 2274 | 2351 | 2429 | 2436 | 2603 | 2823 |
|  | X-angio (n=2) | 2 | 0 | 0 | 0 | 0 | 0 | 0 | 0 | 0 | 0 | 0 |
|  | Total | 5715 | 6068 | 6356 | 6284 | 6362 | 6710 | 6516 | 6781 | 6856 | 7341 | 7872 |
| **Head (n=207,960)** | | | | | | | | | | | | |
|  | X-ray (n=15,059) | 1607 | 1349 | 1348 | 1274 | 1512 | 1628 | 1258 | 1226 | 1405 | 1209 | 1243 |
|  | CT (n=96,108) | 6566 | 6862 | 7348 | 7941 | 8613 | 9323 | 9140 | 9697 | 9931 | 9628 | 11059 |
|  | MRI (n=93,547) | 5687 | 6551 | 6951 | 8250 | 8467 | 8906 | 9178 | 9387 | 9769 | 9485 | 10916 |
|  | NM (n=354) | 41 | 39 | 66 | 41 | 29 | 30 | 27 | 14 | 24 | 26 | 17 |
|  | PET-CT (n=926) | 22 | 18 | 30 | 63 | 117 | 82 | 76 | 110 | 160 | 120 | 128 |
|  | US (n=1035) | 1 | 10 | 13 | 22 | 20 | 30 | 26 | 23 | 35 | 493 | 362 |
|  | X-angio (n=931) | 42 | 79 | 126 | 174 | 139 | 91 | 45 | 39 | 49 | 68 | 79 |
|  | Total | 13,966 | 14,908 | 15,882 | 17,765 | 18,897 | 20,090 | 19,750 | 20,496 | 21,373 | 21,029 | 23,804 |
| **Heart (n=156,547)** | | | | | | | | | | | | |
|  | CT (n=7389) | 460 | 493 | 543 | 509 | 474 | 534 | 769 | 812 | 828 | 991 | 976 |
|  | MRI (n=9898) | 848 | 778 | 741 | 1093 | 671 | 718 | 872 | 952 | 1040 | 1038 | 1147 |
|  | NM (n=33,442) | 3258 | 3407 | 3463 | 3129 | 3233 | 2980 | 2628 | 2771 | 2995 | 2678 | 2900 |
|  | PET-CT (n=4996) | 0 | 0 | 2 | 2 | 5 | 56 | 685 | 1005 | 988 | 1075 | 1178 |
|  | US (n=74,352) | 0 | 0 | 0 | 61 | 98 | 586 | 13333 | 14100 | 15507 | 14645 | 16022 |
|  | X-angio (n=26,470) | 2587 | 2219 | 0 | 0 | 5 | 255 | 4250 | 4090 | 4528 | 4189 | 4347 |
|  | Total | 7153 | 6897 | 4749 | 4794 | 4486 | 5129 | 22,537 | 23,730 | 25,886 | 24,616 | 26,570 |
| **Lower extremity (n=171,007)** | | | | | | | | | | | | |
|  | X-ray (n=115,620) | 12,162 | 11,966 | 11,871 | 12,676 | 10,673 | 9814 | 10,157 | 10,234 | 9619 | 7811 | 8637 |
|  | CT (10,141) | 677 | 844 | 926 | 933 | 959 | 1099 | 1174 | 1085 | 806 | 785 | 853 |
|  | MRI (n=17,982) | 1162 | 1231 | 1323 | 1856 | 1828 | 1944 | 1906 | 1949 | 1939 | 1406 | 1438 |
|  | NM (n=28) | 4 | 3 | 7 | 5 | 2 | 3 | 1 | 1 | 1 | 1 | 0 |
|  | US (n=5757) | 311 | 400 | 404 | 526 | 759 | 706 | 575 | 605 | 641 | 417 | 413 |
|  | X-angio (n=21,479) | 1960 | 1875 | 2143 | 2286 | 2116 | 2038 | 2175 | 1936 | 1911 | 1450 | 1589 |
|  | Total | 16,276 | 16,319 | 16,674 | 18,282 | 16,337 | 15,604 | 15,988 | 15,810 | 14,917 | 11,870 | 12,930 |
| **Neck (n=48,120)** | | | | | | | | | | | | |
|  | X-ray (n=22) | 1 | 0 | 1 | 4 | 5 | 3 | 4 | 2 | 1 | 0 | 1 |
|  | CT (n=3542) | 283 | 284 | 322 | 381 | 413 | 394 | 353 | 246 | 285 | 274 | 307 |
|  | MRI (n=3393) | 176 | 233 | 256 | 283 | 230 | 254 | 278 | 323 | 340 | 463 | 557 |
|  | NM (n=27,618) | 1423 | 1530 | 2416 | 2703 | 2657 | 2695 | 2713 | 2772 | 2872 | 2775 | 3062 |
|  | US (n=13,515) | 88 | 96 | 1218 | 1697 | 1638 | 1325 | 1456 | 1554 | 1688 | 1372 | 1383 |
|  | X-angio (n=30) | 0 | 0 | 3 | 6 | 0 | 1 | 2 | 5 | 5 | 6 | 2 |
|  | Total | 1971 | 2143 | 4216 | 5074 | 4943 | 4672 | 4806 | 4902 | 5191 | 4890 | 5312 |
| **Pelvis (n=56,509)** | | | | | | | | | | | | |
|  | X-ray (n=42,368) | 3214 | 3222 | 3480 | 3728 | 4043 | 4368 | 4675 | 4516 | 4307 | 3235 | 3580 |
|  | CT (n=4006) | 261 | 280 | 267 | 311 | 299 | 386 | 443 | 511 | 412 | 414 | 422 |
|  | MRI (n=7531) | 483 | 443 | 427 | 502 | 548 | 625 | 738 | 845 | 933 | 916 | 1071 |
|  | US (n=1777) | 72 | 87 | 86 | 88 | 84 | 93 | 222 | 298 | 247 | 258 | 242 |
|  | X-angio (n=827) | 47 | 51 | 53 | 72 | 73 | 61 | 82 | 119 | 74 | 103 | 92 |
|  | Total | 4077 | 4083 | 4313 | 4701 | 5047 | 5533 | 6160 | 6289 | 5973 | 4926 | 5407 |
| **Spine (n=135,015)** | | | | | | | | | | | | |
|  | X-ray (n=71,856) | 6666 | 6732 | 6948 | 6918 | 6615 | 7592 | 7030 | 6545 | 5956 | 4936 | 5918 |
|  | CT (n=13,831) | 868 | 981 | 1143 | 1174 | 1562 | 1914 | 2197 | 1905 | 696 | 598 | 793 |
|  | MRI (n=38,756) | 2431 | 2718 | 2884 | 3569 | 3325 | 3752 | 3848 | 4063 | 3982 | 3815 | 4369 |
|  | NM (n=1) | 0 | 0 | 0 | 0 | 0 | 0 | 0 | 1 | 0 | 0 | 0 |
|  | US (n=54) | 0 | 0 | 0 | 1 | 26 | 23 | 4 | 0 | 0 | 0 | 0 |
|  | X-angio (n=10,517) | 842 | 766 | 845 | 919 | 817 | 828 | 940 | 1147 | 1131 | 1093 | 1189 |
|  | Total | 10,807 | 11,197 | 11,820 | 12,581 | 12,345 | 14,109 | 14,019 | 13,661 | 11,765 | 10,442 | 12,269 |
| **Thorax (n=283,017)** | | | | | | | | | | | | |
|  | X-ray (n=214,232) | 22,683 | 22,944 | 22,177 | 21,786 | 20,420 | 19,951 | 19,144 | 18,854 | 18,684 | 13,888 | 13,701 |
|  | CT (n=57,866) | 3399 | 3545 | 3726 | 4179 | 4642 | 4983 | 5563 | 5627 | 6390 | 7664 | 8148 |
|  | MRI (n=1157) | 46 | 64 | 77 | 63 | 104 | 120 | 124 | 131 | 154 | 129 | 145 |
|  | NM (n=3977) | 289 | 374 | 386 | 391 | 377 | 392 | 349 | 329 | 385 | 350 | 355 |
|  | US (n=2909) | 27 | 38 | 77 | 121 | 184 | 206 | 283 | 286 | 308 | 575 | 804 |
|  | X-angio (n=2876) | 156 | 141 | 157 | 202 | 241 | 239 | 219 | 220 | 223 | 336 | 742 |
|  | Total | 26,600 | 27,106 | 26,600 | 26,742 | 25,968 | 25,891 | 25,682 | 25,447 | 26,144 | 22,942 | 23,895 |
| **Trunk (n=40,824)** | | | | | | | | | | | | |
|  | X-ray (n=7) | 6 | 0 | 0 | 0 | 0 | 0 | 0 | 0 | 1 | 0 | 0 |
|  | CT (n=34,832) | 1716 | 1996 | 2423 | 2610 | 2821 | 3232 | 3628 | 4135 | 3769 | 3984 | 4518 |
|  | US (n=5206) | 172 | 205 | 253 | 349 | 555 | 625 | 573 | 683 | 577 | 574 | 640 |
|  | X-angio (n=779) | 0 | 0 | 0 | 0 | 0 | 0 | 0 | 83 | 238 | 209 | 249 |
|  | Total | 1894 | 2201 | 2676 | 2959 | 3376 | 3857 | 4201 | 4901 | 4585 | 4767 | 5407 |
| **Upper extremity (n=154,927)** | | | | | | | | | | | | |
|  | X-ray (n=112,076) | 8956 | 8837 | 9337 | 9941 | 10172 | 11377 | 11436 | 10883 | 10666 | 10050 | 10421 |
|  | CT (n=7397) | 294 | 437 | 587 | 683 | 703 | 823 | 779 | 805 | 786 | 682 | 818 |
|  | MRI (n=6266) | 455 | 465 | 464 | 565 | 610 | 629 | 661 | 629 | 623 | 538 | 627 |
|  | US (n=9877) | 284 | 319 | 230 | 251 | 661 | 783 | 1355 | 1588 | 1581 | 1386 | 1439 |
|  | X-angio (n=19,311) | 1080 | 1070 | 1096 | 1560 | 1690 | 1958 | 2110 | 2323 | 2245 | 2031 | 2148 |
|  | Total | 11,069 | 11,128 | 11,714 | 13,000 | 13,836 | 15,570 | 16,341 | 16,228 | 15,901 | 14,687 | 15,453 |
| **Whole body (n=32,144)** | | | | | | | | | | | | |
|  | X-ray (n=6578) |  | 1 | 247 | 732 | 707 | 675 | 736 | 904 | 785 | 830 | 961 |
|  | CT (n=8000) | 233 | 320 | 429 | 542 | 705 | 840 | 1002 | 1091 | 1027 | 883 | 928 |
|  | MRI (n=1786) | 217 | 134 | 111 | 111 | 99 | 107 | 121 | 151 | 217 | 262 | 256 |
|  | NM (n=8569) | 875 | 879 | 896 | 881 | 775 | 733 | 755 | 669 | 628 | 652 | 826 |
|  | PET-CT (n=985) | 0 | 0 | 0 | 0 | 0 | 0 | 91 | 171 | 194 | 241 | 288 |
|  | US (n=6226) | 0 | 0 | 0 | 0 | 62 | 754 | 1022 | 994 | 1106 | 1056 | 1232 |
|  | Total | 1325 | 1334 | 1683 | 2266 | 2348 | 3109 | 3727 | 3980 | 3957 | 3924 | 4491 |

^a^CT: computed tomography.

^b^MRI: magnetic resonance imaging.

^c^NM: nuclear medicine.

^d^PET: positron emission tomography.

^e^US: ultrasonography.

^f^X-angio: x-ray angiography.
