# Supplementary material for: Natural Language Processing and Graph Theory: Making Sense of Imaging Records in a Novel Representation Frame
Source: JMIR Med Inform. 2022 Dec 21;10(12):e40534. doi: 10.2196/40534 (PMC9813822; doi:10.2196/40534)
Supplement: Multimedia Appendix 4 [file medinform_v10i12e40534_app4.docx]

## Table S3. Average *F*_1_-score of the 5-fold cross-validation on 2646 reports.

| Variable | Average *F*_1_-score (95% CI) |
| --- | --- |
| Date | 0.89 (0.87-0.92) |
| No previous | 0.97 (0.96-0.99) |
| Today | 0.87 (0.80-0.94) |
| Overall | 0.93 (0.91-0.93) |
