# Supplementary material for: Natural Language Processing and Graph Theory: Making Sense of Imaging Records in a Novel Representation Frame
Source: JMIR Med Inform. 2022 Dec 21;10(12):e40534. doi: 10.2196/40534 (PMC9813822; doi:10.2196/40534)
Supplement: Multimedia Appendix 5 [file medinform_v10i12e40534_app5.docx]

**Table S4.** Temporal reference of the reports (n=1,514,220) per modality.

| Modality | Previous, % | No previous, % | Missing, % |
| --- | --- | --- | --- |
| CT^a^ | 58.78 | 29.62 | 11.60 |
| X-ray | 51.25 | 19.15 | 29.60 |
| MRI^b^ | 59.25 | 18.64 | 22.11 |
| Mammography | 79.03 | 13.42 | 7.55 |
| X-angiography | 12.31 | 4.46 | 83.23 |
| Ultrasonography | 41.36 | 21.49 | 37.15 |
| PET^c^-CT | 62.92 | 26.76 | 10.32 |
| NM^d^ | 50.42 | 14.84 | 34.74 |

^a^CT: computed tomography.

^b^MRI: magnetic resonance imaging.

^c^PET: positron emission tomography.

^d^NM: nuclear medicine.

**Table S5.** Temporal reference of the reports (n=1,514,220) per body region.

| Body region | Previous, % | No previous, % | Missing, % |
| --- | --- | --- | --- |
| Whole body | 42.33 | 21.05 | 36.62 |
| Upper extremity | 33.66 | 16.39 | 49.95 |
| Trunk | 76.09 | 16.16 | 7.75 |
| Thorax | 69.79 | 20.92 | 9.29 |
| Pelvis | 49.75 | 15.71 | 34.53 |
| Neck | 26.22 | 10.43 | 63.35 |
| Lower extremity | 39.16 | 18.32 | 42.52 |
| Heart | 14.13 | 12.94 | 72.94 |
| Head | 54.80 | 31.58 | 13.62 |
| Breast | 78.44 | 13.45 | 8.11 |
| Abdomen | 49.26 | 30.51 | 20.23 |
| Spine | 53.05 | 16.73 | 30.21 |

**Table S6**. Temporal connectivity of the reports (n=1,514,220) at the modality level.

| Referencing modalities | Referenced modalities, % | | | | | | | |
| --- | --- | --- | --- | --- | --- | --- | --- | --- |
|  | X-ray | CT^a^ | MRI^b^ | Mammography | NM^c^ | PET^d^-CT | US^e^ | X- angio^f^ |
|  |  |  |  |  |  |  |  |  |
| X-ray | 72.51 | 15.79 | 2.75 | 0.03 | 0.45 | 0.07 | 4.04 | 4.35 |
| CT | 21.88 | 60.55 | 9.46 | 0.05 | 1.14 | 0.21 | 4.81 | 1.91 |
| MRI | 17.57 | 23.1 | 52.39 | 0.75 | 0.39 | 0.15 | 3.79 | 1.05 |
| Mammography | 4.2 | 0.84 | 2.29 | 57.94 | 0.09 | 0.01 | 34.52 | 0,.2 |
| NM | 18.79 | 33.90 | 8.29 | 0,13 | 33.74 | 0.27 | 3.77 | 1.11 |
| PET-CT | 2.47 | 23.62 | 45.06 | 0.00 | 4.84 | 22.33 | 1.48 | 0.20 |
| US | 11.10 | 16.95 | 4.85 | 22.08 | 0.41 | 0.03 | 43.63 | 0.95 |
| X-angio | 39.75 | 20.94 | 9.30 | 0.00 | 1.00 | 0.13 | 2.35 | 26.52 |

^a^CT: computed tomography.

^b^MRI: magnetic resonance imaging.

^c^NM: nuclear medicine.

^d^PET: positron emission tomography.

^e^US: ultrasonography.

^f^X-angio: x-ray angiography.
